# CT-based and morphological comparison of glenoid inclination and version angles and mineralisation distribution in human body donors

**DOI:** 10.1186/s12891-021-04660-4

**Published:** 2021-10-05

**Authors:** Nabil Serrano, Marc Kissling, Hannah Krafft, Karl Link, Oliver Ullrich, Florian M. Buck, Sandra Mathews, Steffen Serowy, Dominic Gascho, Patrick Grüninger, Paolo Fornaciari, Samy Bouaicha, Magdalena Müller-Gerbl, Frank-Jakobus Rühli, Elisabeth Eppler

**Affiliations:** 1 Institute of Evolutionary Medicine (IEM), University of Zurich, Zurich, Switzerland; 2 Musculoskeletal Research, Department of Biomedicine, University of Basel, Basel, Switzerland; 3 Division of Gross Anatomy, Institute of Anatomy, University of Zurich, Zurich, Switzerland; 4 Anatomy, University of Fribourg, Fribourg, Switzerland; 5 Medical Radiology Institute, Schulthess Clinic, Zurich, Switzerland; 6 Clinic of Neuroradiology, University Hospital of Magdeburg, Magdeburg, Germany; 7 Institute of Forensic Medicine, University of Zurich, Zurich, Switzerland; 8 Department of Surgery, Limmattal Hospital, Zurich, Switzerland; 9 Department of Orthopaedics, Balgrist University Hospital, Zurich, Switzerland; 10 Department of Orthopaedic Surgery and Traumatology, University Hospital Fribourg, Fribourg, Switzerland; 11 Institute of Anatomy, University of Bern, Bern, Switzerland

**Keywords:** Shoulder joint, 3D-CT, CT-OAM, Inclination angle, Glenoid anteversion, Glenoid retroversion, Bone mineralization

## Abstract

**Background:**

For optimal prosthetic anchoring in omarthritis surgery, a differentiated knowledge on the mineralisation distribution of the glenoid is important. However, database on the mineralisation of diseased joints and potential relations with glenoid angles is limited.

**Methods:**

Shoulder specimens from ten female and nine male body donors with an average age of 81.5 years were investigated. Using 3D-CT-multiplanar reconstruction, glenoid inclination and retroversion angles were measured, and osteoarthritis signs graded. Computed Tomography-Osteoabsorptiometry (CT-OAM) is an established method to determine the subchondral bone plate mineralisation, which has been demonstrated to serve as marker for the long-term loading history of joints. Based on mineralisation distribution mappings of healthy shoulder specimens, physiological and different CT-OAM patterns were compared with glenoid angles.

**Results:**

Osteoarthritis grades were 0-I in 52.6% of the 3D-CT-scans, grades II-III in 34.3%, and grade IV in 13.2%, with in females twice as frequently (45%) higher grades (III, IV) than in males (22%, III). The average inclination angle was 8.4°. In glenoids with inclination ≤10°, mineralisation was predominantly centrally distributed and tended to shift more cranially when the inclination raised to > 10°. The average retroversion angle was − 5.2°. A dorsally enhanced mineralisation distribution was found in glenoids with versions from − 15.9° to + 1.7°. A predominantly centrally distributed mineralisation was accompanied by a narrower range of retroversion angles between − 10° to − 0.4°.

**Conclusions:**

This study is one of the first to combine CT-based analyses of glenoid angles and mineralisation distribution in an elderly population. The data set is limited to 19 individuals, however, indicates that superior inclination between 0° and 10°-15°, and dorsal version ranging between − 9° to − 3° may be predominantly associated with anterior and central mineralisation patterns previously classified as physiological for the shoulder joint. The current basic research findings may serve as basic data set for future studies addressing the glenoid geometry for treatment planning in omarthritis.

**Supplementary Information:**

The online version contains supplementary material available at 10.1186/s12891-021-04660-4.

## Background

Osteoarthritis is one of the most common diseases worldwide, causing pain and severe restrictions of the range of motion and quality of life, particularly in the elderly [39, 52, 54]. Progressive disease causes extensive morphological changes in articular cartilage and subchondral bone, which may result in narrowed joint gap, subchondral sclerosis, osteophytes and deformation of the glenoid including alterations of glenoid angles. An ideal range is not clearly defined to date, but the geometry of the glenoid may predispose for alterations [15, 17, 36, 39, 55]. To achieve an optimal treatment outcome, e.g., positioning of the prosthetic glenoid component, a profound knowledge on optimal geometry, particularly the glenoid angles, and bone mineralisation is essential [3, 15–17, 29, 33, 39].

More than 30 years ago, Computed Tomography Osteoabsorptiometry (CT-OAM) was established [38] to determine the mineralisation distribution of the subchondral bone plate, which has been demonstrated as suitable marker for the long-term loading history of manifold articulations in animal species and humans, e.g. [9, 32, 40], including healthy [18, 28, 56–58], and osteoarthritic [46, 49] shoulder joints. In the present study, we used CT-based methods to compare the mineralisation distribution of the glenoid with inclination and version angles in order to expand the data set on physiological and different geometry and mineralisation patterns of the shoulder joint as an entity [58].

For that purpose, measurements of both, glenoid and humeral head were performed by 3D-CT multiplanar reconstructions to compare the present data with those from previous studies [35, 56–58]. Further, osteoarthritic alterations were graded using 3D-CT reconstructions. As a next step, inclination and version angles were individually compared based on CT-measurements with the mineralisation distribution, and discrepancies between angles and mineralisation patterns further analysed for morphological and radiological signs of osteoarthritis. Finally, a range of inclination and ante−/retroversion angles was defined, which contained the majority of physiological mineralisation distribution patterns as previously defined [45].

## Methods and material

### Body donors

The study was performed on shoulder specimens from ten female and nine male body donors with an average age of 81.5 years (± 11.3 years, range: 60–98 years, median: 85 years) from the body donation programme of the Institute of Anatomy of the University of Zurich [21] according to the Federal Act on Research involving Human Beings (Human Research Act, HRA) of 1 January 2014 [20] and the Guidelines of the Swiss Academy of Medical Sciences, updated 2014 [22, 23] as described [35]. The bodies served for the dissection course of 2nd year medical bachelor students during the curricular years 2013/2014, 2014/2015, and 2015/2016.

### Dissection and morphological classification of the shoulder specimens

**Fig. 1:**
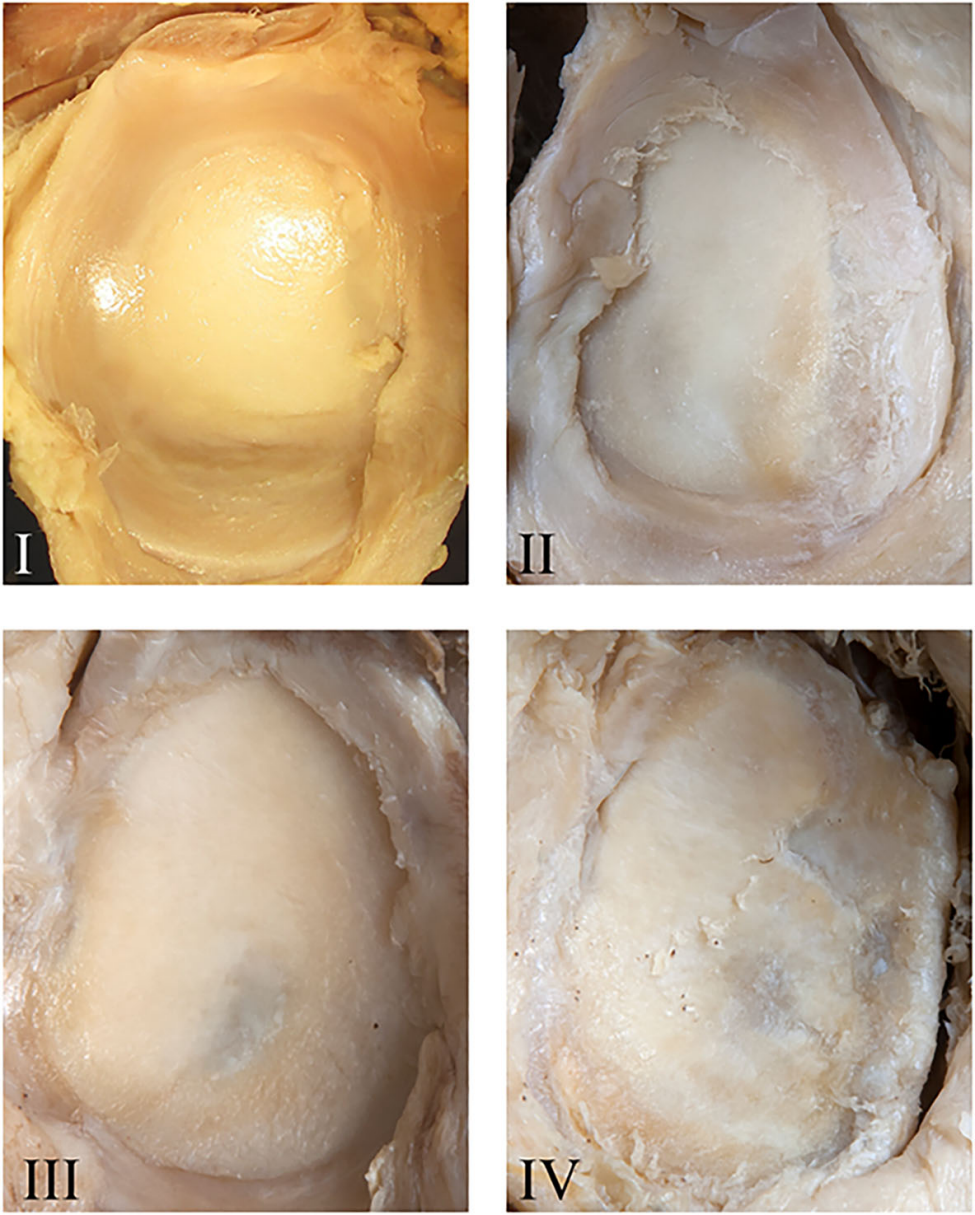
Representative images of osteoarthritis grades I, II, III and IV in dissected glenoids

**Fig. 2:**
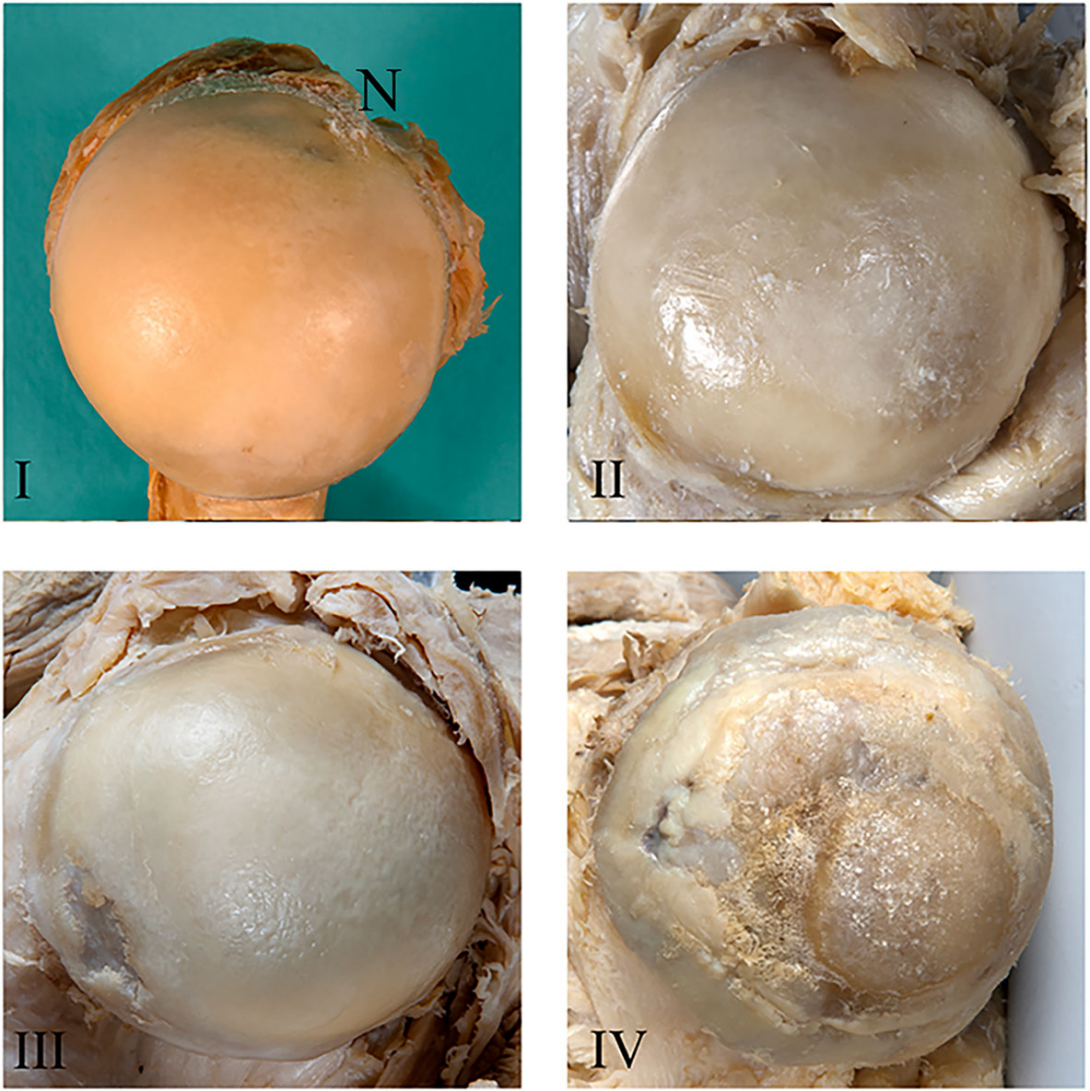
Representative images of osteoarthritis grades I, II, III and IV in dissected humeral heads. (I) N points to the notch in the humeral head formed by the tendon of the long head of the biceps used as a reference for CT-OAM and morphological inspections

The gleno-humeral joint was exposed by the delto-pectoral approach as described [35]. We deliberately did not exclude specimens with signs of pathologies to create a representative data set for an aged study population. Glenoid and humeral head were graded by three experienced shoulder surgeons using the same quadrants as for CT-OAM (see below) according to the Outerbridge classification [2, 47, 51], adapted for body donor specimens (Figs. 1 and 2): Grades 0 (normal cartilage) and I were merged to grade I since grade I chondral lesions characterized by softening and swelling [51], often requiring tactile feedback [47] were difficult to differentiate in formalin-fixated donor samples. We also assumed grade I lesions in this aged cohort as physiological. Grade II lesions were characterized by partial thickness defects with visually detectable softening, blistering, small fissures and fibrillations without contact to the subchondral bone, grade III by larger cartilage fissures and deep ulcerations, sometimes reaching the subchondral bone, and grade IV by erosions exposing the subchondral bone [2, 47, 51]. Discrepancies were discussed by an experienced anatomy lecturer and two young physicians for final decision.

### CT scan acquisition

CT scans were acquired intended for virtual teaching prior to the dissection course at the Institute of Forensic Medicine of the University of Zurich using a 128-slice CT scanner (Somatom Definition Flash, Siemens Healthcare, Forchheim, Germany). The scan parameters were 120 kV and 500 reference mAs using dose modulation CAREdose4D™ (Siemens Healthcare) as described [14]. Raw data were reconstructed with a slice thickness of 0.6 mm and an increment of 0.4 mm using a hard kernel (B60) for 3D-analysis and a soft kernel (B30) for CT-OAM. The size of the reconstruction field of view was adjusted for separate reconstructions of left and right shoulder. DICOM files were transferred to OSIRIX™ medical imaging viewer (Pixmeo SARL, Bernex, Switzerland).

### 3D-CT reconstruction of the gleno-humeral joint

The osseous window enabled to eliminate all soft tissue. To obtain an unobstructed view of each articular surface, either humerus, or scapula and clavicle were extracted. For optimal selection and extraction of a bone, the region of interest segmentation was set from 300 to 2′000 Hounsfield units (HU). The volume content was erased, and the 3D volume rendering option rendered a 3D image. The resulting articular surface was displayed with two different pre-sets, i.e., “Bone CT glossy” and “Basic with low contrast”. Data were exported as DICOM-series at highest quality scale for best image, for the glenoid in a sagittal view and for the humeral head in two 180° orientations, i.e., anterior and lateral view (horizontal and vertical).

### 3D-CT-based analyses of joint surfaces

**Fig. 3:**
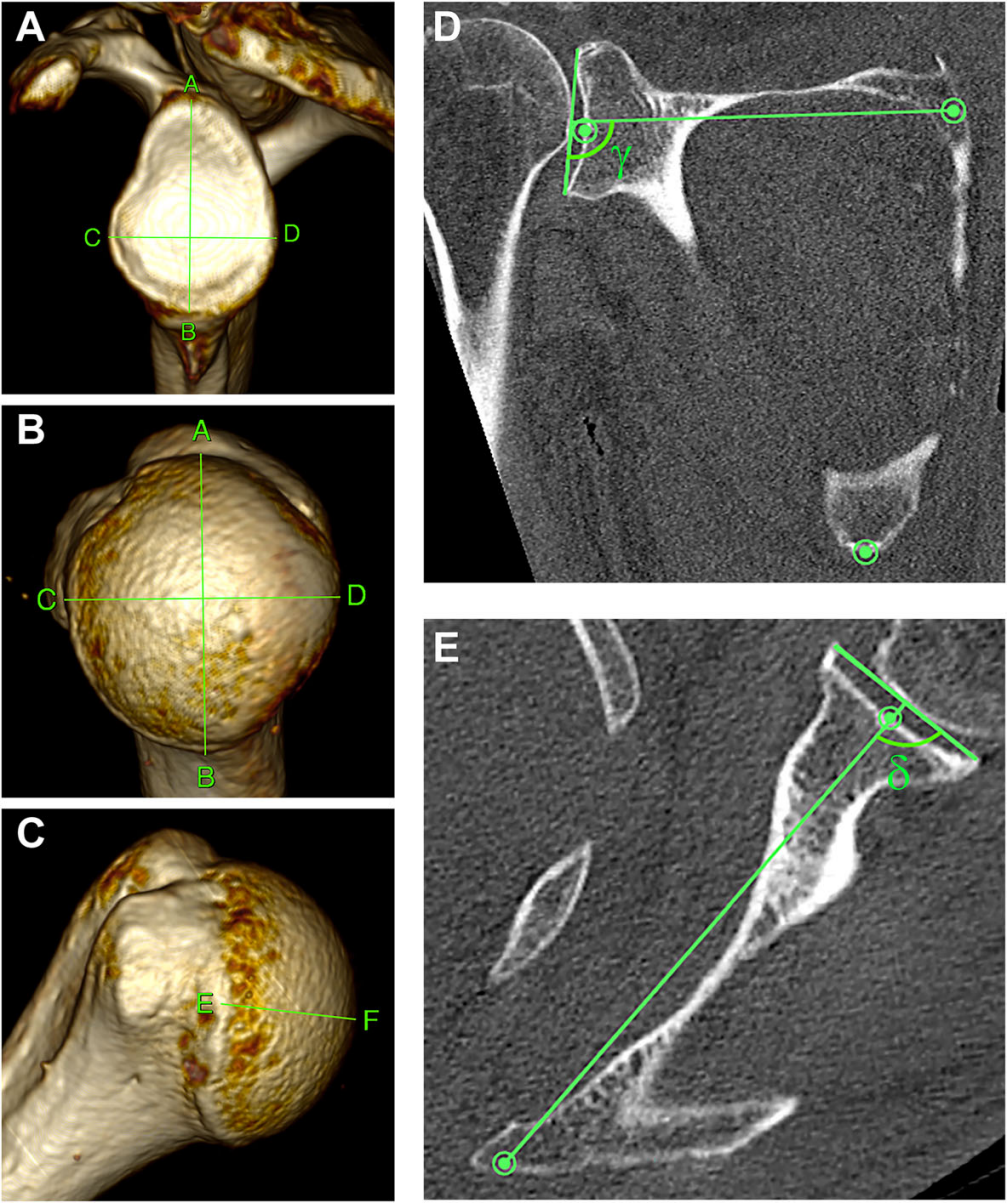
2D-CT images of 3D-CT reconstructions of the articular surfaces of a left shoulder. **A** Height and width of the glenoid measured as described [35]: a line drawn from the most cranial (**A**) to the most caudal point (**B**) of the glenoid cavity, and another line from the most ventral (**C**) to the most dorsal point (**D**) of the glenoid rim. **B** Height and width of the humeral head measured using established landmarks [26]: the greater tubercle as reference to place the uppermost point (**A**) at the edge of the smooth articular surface and a perpendicular line was drawn to the lowest (**B**) point. **C** The depth of the humeral head measured using established landmarks [26]: the lesser tubercle as reference for the most anterior point (**F**), from this, a perpendicular line was drawn to the most posterior point (**E**) of the smooth articular surface. **D** The inclination angle α was determined in the coronal plane using reference points (green) as described [7, 35]: a line was drawn through the root of the scapular spine to the midpoint of the glenoid cavity, and a second line through the most cranial and caudal points (see **A**, reference points **A** and **B**) of the glenoid rim. Angle γ was measured between the axes of these two lines in caudal direction, and the inclination angle α calculated: α = γ - 90°. **E** The ante−/retroversion angles β were determined in the transversal plane using reference points (green) as described [13, 35]: a line was drawn through the root of the scapular spine to the midpoint of the glenoid cavity, and a second line through the most posterior and anterior points (see **A**, reference points **C** and **D**) of the glenoid rim. Angle δ was measured between the axes of these two lines in sagittal direction and angle β calculated: β = δ - 90°

In order to compare the data set with previous studies, height and width of the joint surfaces were measured. The glenoid (Fig. 3A) was measured according to Martin and Saller [34] as commonly used intraoperatively and adapted to our specimens [35]. Height, width (Fig. 3B) and depth (Fig. 3C) of the humeral head were measured using established referencing points, whereby osteophytes were strictly avoided to prevent overestimation of the surface area [26].

### 3D-CT measurements of inclination and ante−/retroversion angles

In the 3D-multiplanar reconstruction mode (Fig. 3D,E), a plane of the scapula was defined by the midpoint of the root of the scapular spine, the center of the glenoid, and the most distal point of the inferior scapula angle as described [6, 35]. Inclination was determined in the coronal plane according to Churchill et al. [7]: The angle γ was measured (Fig. 3D) and the inclination angle α calculated by the formula: α = γ - 90° as described [35]. Positive angles describe superior inclination [15]. The ante−/retroversion was determined in the transversal plane according to Friedman method [13]: The angle δ was measured (Fig. 3E) and the version angle β calculated by the formula: β = δ - 90° as described [35]. Negative values indicate retroversion, positive values anteversion [15, 35].

### 3D-CT –based analyses of osteoarthritis signs

For analysis of osteoarthritis signs in the gleno-humeral joints using 3D-CT (Fig. 3), an osteoarthritis grading score was created based on the Kellgren-Lawrence Score [25] and adapted to body donors considering the Larsen [30] and Samilson and Prieto [44] radiological classifications (Table 1).

**Table 1:** Grading score of 3D-CT scans of the gleno-humeral joint

Points	Kellgren-Lawrence Score [25]	Samilson and Pietro Classification [44]	Larsen Classification [30]
0	absence of signs of osteoarthritis	no exostosis	normal conditions, marginal bone deposits
1	definite osteophytes and possible joint space narrowing	inferior humeral and/or glenoid exostosis < 3 mm in height	definite early abnormality, erosion and joint space narrowing present
2	moderate multiple osteophytes, definite narrowing of joint space, some sclerosis	inferior humeral and/or glenoid exostosis measuring 3–7 mm, and slight gleno-humeral irregularity	medium to severe destructive abnormality, erosion and joint space narrowing present
3	large osteophytes, marked narrowing of joint space, severe sclerosis	inferior humeral and/or glenoid exostosis measuring > 7 mm, gleno-humeral joint narrowing and sclerosis	mutilating abnormality, gross bony destruction, dislocation and ankylosis
Maximum 9 points	Maximum 3 points	Maximum 3 points	Maximum 3 points

Established osteoarthritis classifications were considered [25, 30, 44]. Grade 0: no osteoarthritis signs, grade 1: 1–2 points, grade 2: 3–4 points, grade 3: 5–7 points, grade 4: 8–9 points

### Bone mineralisation analysis by CT-osteoabsorptiometry (CT-OAM)

**Fig. 4:**
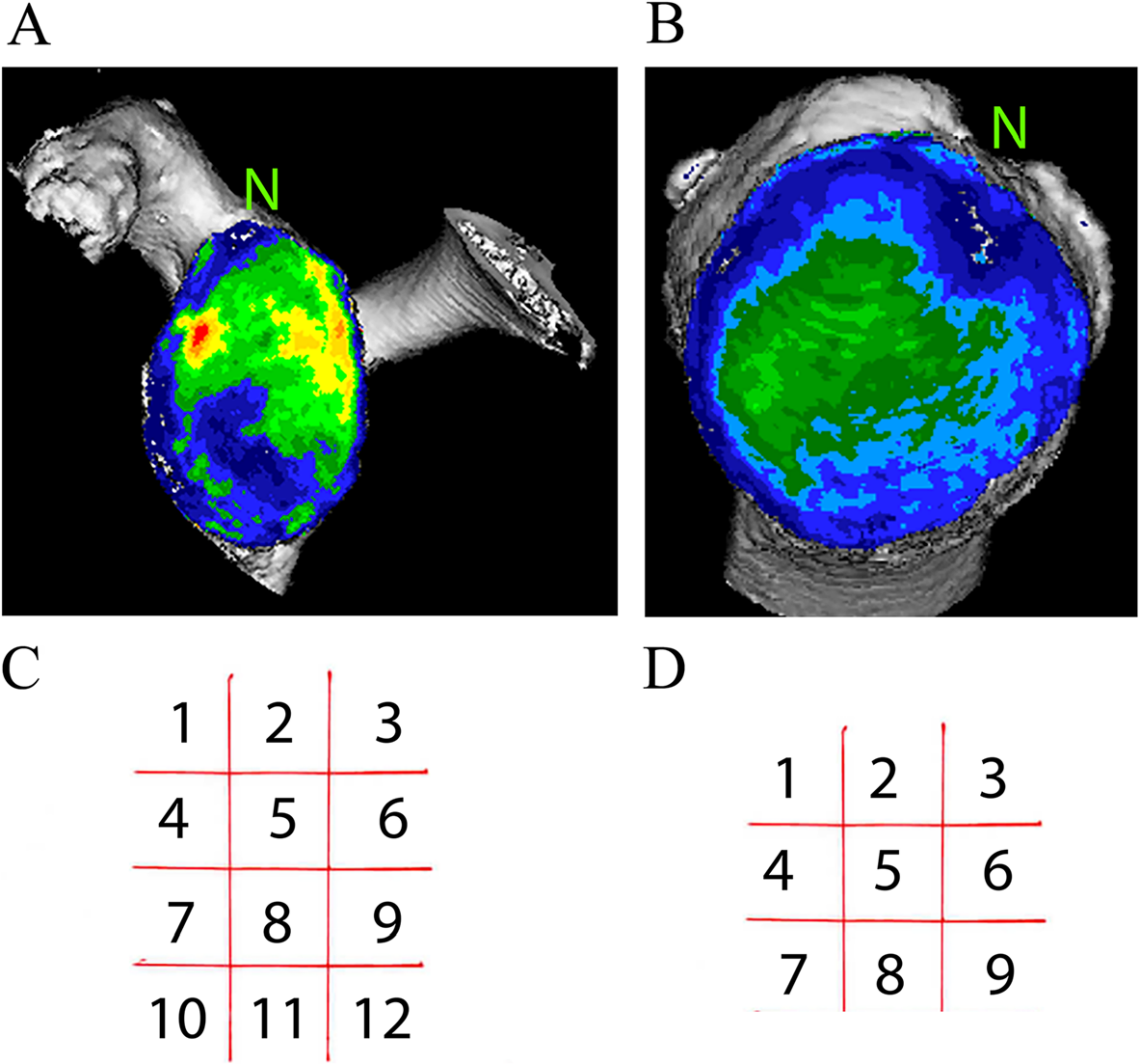
Representative CT-OAM images showing mineral distribution of (**A**) glenoid, and (**B**) humeral head. CT-OAM false colour-encoded (black > red > orange > yellow > bright green > dark green > blue > violet, see: [57]) and superimposed on the identical 3-D-reconstructed joint. N points (**A**) to the most superior point of the glenoid, and (**B**) to notch in the humeral head formed by the tendon of the long head of biceps. (**C**, **D**) A grid was superimposed over the CT-OAM and morphology images to separate the articular surface in quadrants of glenoid (**C**) and humeral head (**D**) similar to previous work [57, 58]

CT-OAM analyses were performed as established for healthy shoulders [45, 56–58]. In brief, DICOM data sets were analysed using an image analysis system (ANALYZE, version 7.4, Biomedical Imaging Resource, Mayo Foundation, Rochester, MN, USA). The subchondral bone plate was isolated by segmentation of CT scans. By means of maximum intensity projection, for each pixel until a depth of 3 mm, values with the highest density were projected onto the surface. Threshold values were selected according to previous studies to be < 200 to > 1200 HU [28]. To display the mineralisation distribution, data were false colour-encoded and superimposed on the identical 3D-reconstructed joint (Fig. 4A,B) as described [57]. Areas of highest mineralisation distribution were identified as “central”, “cranial”, “caudal”, “ventral” or “dorsal” by quadrants of the articular surfaces (Fig. 4C,D) similar to previous work [57, 58]. The mineralisation distribution patterns (Fig. 4) were compared with those of previous studies from our group in healthy shoulders for typical criteria of physiological distributions [28, 45], i.e., predominantly central and anterior > posterior as described [45]. Mineralisation patterns differing from these physiological patterns were classified as “different” or “non-physiological”, respectively.

### Comparison of inclination and ante−/retroversion angles with mineralisation distribution

As a next step, the mineralisation distribution was compared with inclination and version angles. Samples were grouped as described (Table 2).

**Table 2:** Comparison of samples based on CT-mineralisation distribution and glenoid angles

CT-based measurement	physiological: central and anterior > dorsal	Different mineralisation pattern
Inclination angle ≤10° (15°)	*positive*	*negative*
Ante−/retroversion angle ranging between −8° to −4°	*positive*	*negative*

**Fig. 5:**
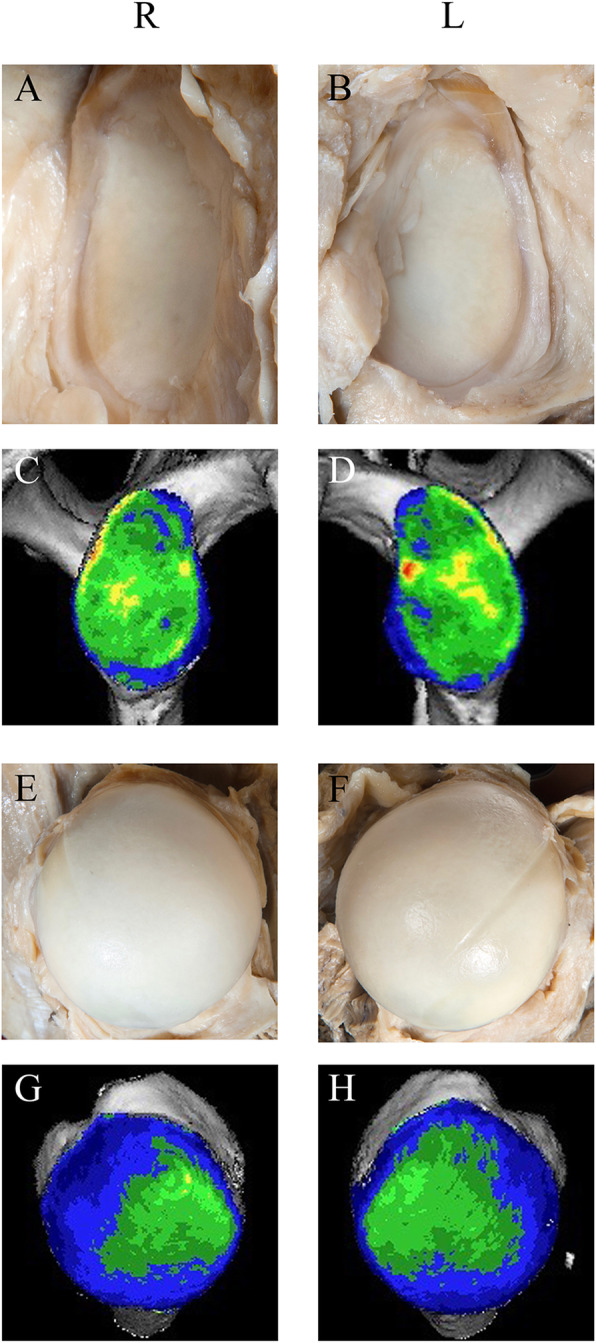
Glenoid and humeral head specimens of a 69 years old female. Inclination: R 10.7°, L 15.9°; retroversion: R -7.6, L − 9°. Note homogenous mineralisation distribution with anterior maxima of the glenoid (**D**), mirrored by intact cartilage (**B**) and some spots on the glenoid rim (**C**) with some cartilage erosions (**A**). Monocentric mineralisation pattern of the humeral head (**G**, **H**) is accompanied by healthy cartilage (**E**, **F**). Note pronounced (L > R) chondral print

Glenoid samples compared by their physiological mineralisation pattern (see Figs. 4 and 5) according to Schulz et al. [45], and glenoid angles defined in the order of magnitude of current literature [12, 15, 16, 19, 24, 35, 41, 42, 48]. In selected cases, physiological inclination was expanded to 15° in this aged cohort. *“Positive”* defined as physiological CT-OAM-patterns and angles within our defined physiological range or, as different CT-OAM-patterns and angles outside this range. *“Negative”* defined as physiological CT-OAM patterns and angles outside this range or, as different CT-OAM patterns and angles within the range

### Data analysis and statistics

Quantitative measurements were expressed as absolute values, mean (± standard deviation) and median. Qualitative variables were compared as described and the positive results calculated as percentage.

## Results

### Study population and 3D-CT specimen characteristics

Glenoid and humeral head sizes from ten female and nine male body donors were similar to our ([35] with further references) and other published data [26] so that we considered the cohort as representative. Similarly, all parameters were smaller in females (Supplement 1). In 52.6% of CT scans, osteoarthritis grades 0-I were observed, in 34.3% grades II-III, and in 13.2% grade IV (Table 3). Respecting potential post-mortem changes, data analysis was restricted to glenoid malformation (*n* = 8), osteophytes (*n* = 14), and narrowed joint gap (*n* = 11) in both sides together.

**Table 3:** Summarised 3D-CT-based radiological osteoarthritis grades

Grade 0	Grade I	Grade II	Grade III	Grade IV
11/38 (28.9%)	9/38 (23.7%)	5/38 (13.2%)	8/38 (21.1%)	5/38 (13.2%)

Grading of gleno-humeral osteoarthritis based on Kellgren-Lawrence Score [25], Larsen classification [30], and Samilson and Prieto radiological classification [44] (Table 1)

Higher osteoarthritis grades were detected twice as frequently in females, i.e., 9/20 shoulders (45%, grade III-IV) versus 4/18 (22%, grade III) in males (Table 4).

**Table 4:** Summarised 3D-CT-based radiological osteoarthritis grades sorted by sex

	Grade 0	Grade I	Grade II	Grade III	Grade IV
male	3/18	9/18	2/18	4/18	0/18
female	8/20	0/20	3/20	4/20	5/20

Grading of gleno-humeral osteoarthritis sorted by sex based on Kellgren-Lawrence Score [25], Larsen classification [30], and Samilson and Prieto radiological classification [44] (Table 1)

### 3D-CT-measurements of glenoid angles

The average inclination angle from male and female donors combined was 8.4° ± 4.9° (range 0.5° to + 19.8°, median: 7.8°), left 8.6° ± 5.0° (range 0.5° to + 19.8°, median: 7.9°), and right 8.2° ± 5.0° (range 1.0° to + 19.4°, median: 7.8°). The average ante−/retroversion angle from male and female donors combined was − 5.2° ± 4.2° (range − 15.9° to + 3.8°, median: − 4.9°), left − 4.8° ± 4.8° (range − 15.9° to + 3.8°, median: − 3.8°), right − 5.6° ± 3.7° (range − 14.3° to + 1.6°, median: -5.8°).

### CT-OAM analyses of glenoid mineralisation patterns

**Fig. 6:**
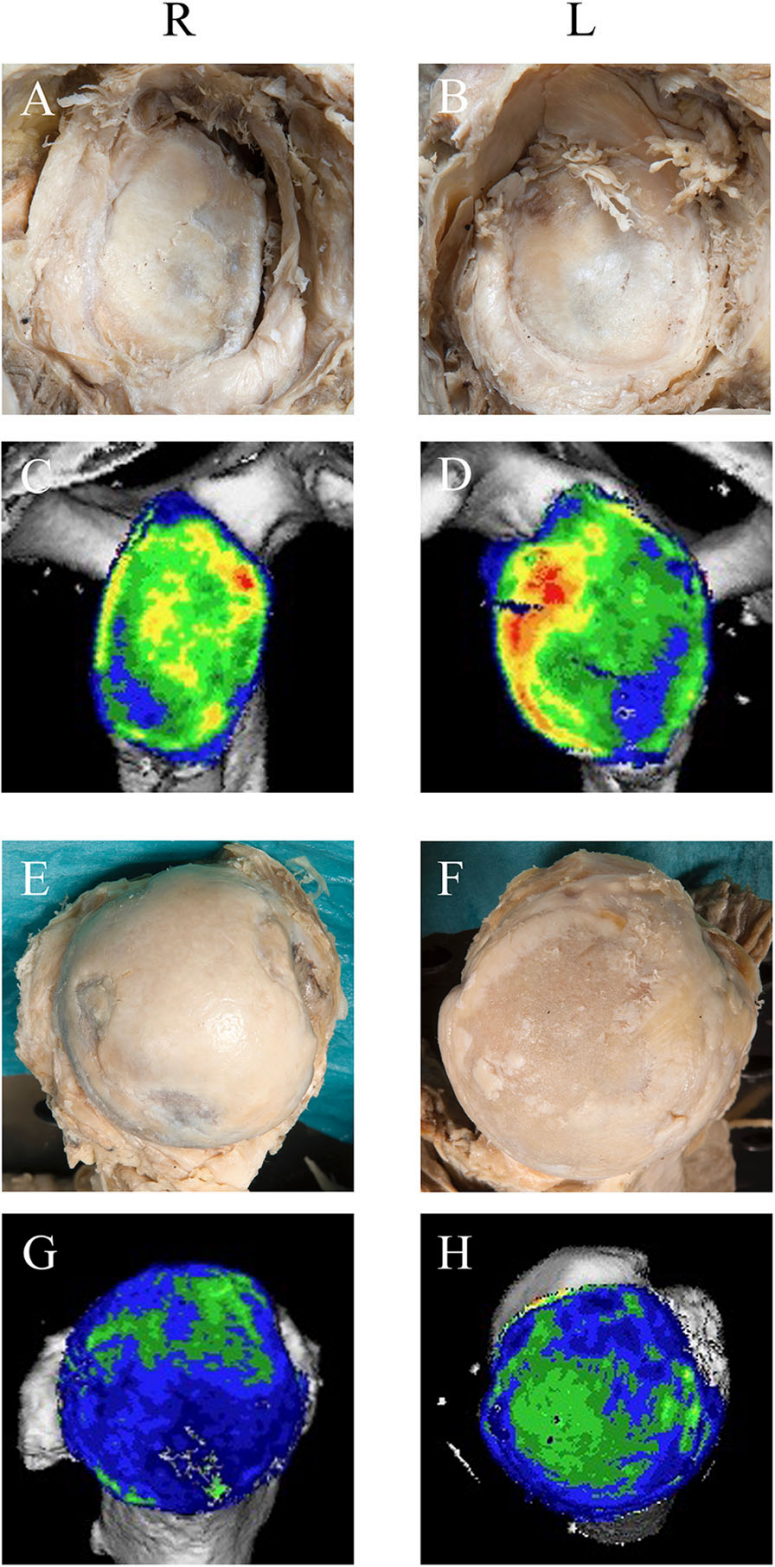
Glenoid and humeral head specimens of a 74 years old male with pronounced signs of osteoarthritis (**A**,**B**). On the right (R), inclination (7.1°) and retroversion (− 2.7°) with homogenously distributed mineralisation and a moderate caudal shift (**C**). On the left (L), inclination (10.7°) and retroversion (− 9.5°) combined with antero-caudal enhanced mineralisation (**D**). Both humeral heads with pronounced osteoarthritic defects grade IV in the dissected specimens (**E**, **F**), without (**G**) or only partial (**H**) congruency to mineralisation distribution

In 19 left specimens, glenoids were classified as physiological mineralisation distribution (compare Figs. 4A and 5D) in vertical (eleven cases), and horizontal orientation (nine cases) according to published patterns [45]. Among the differing mineralisation patterns, enlarged areas of mineralisation were visible cranial in five cases (63%), caudal in three cases (37%) (Fig. 6D), posterior in nine (90%), and anterior in one (10%) case (Fig. 6D). The corresponding humeral head showed grade IV erosion of the articular surface, which was partly congruent (Fig. 6F,H) to areas of mineralisation distribution considered as physiological (compare Figs. 4B and 5H), but partly to areas of reduced mineralisation distribution, particularly in the periphery.

In 19 right specimens, physiological mineralisation distributions were detected in twelve cases in vertical (Fig. 5C) and in seven cases horizontal orientation. Among the differing patterns, mineralisation was enhanced in five cases (71%) more cranial, and in two (29%) more caudal. Among the twelve differing patterns in horizontal orientation, in nine (75%) individuals, the mineralisation was dorsally enhanced. In three cases (25%), a different pattern was observed, e.g. diagonal (one case, not shown) or evenly distributed with moderate caudal shift (2 cases, see Fig. 6C). The corresponding humeral head showed pronounced osteoarthritic defects grade IV at the periphery of the articular surface, while the mineralisation was distributed predominantly centrally with some scattered maxima and, in the periphery, minima (Fig. 6E,G).

### Comparison of inclination angles and CT-OAM

**Fig. 7:**
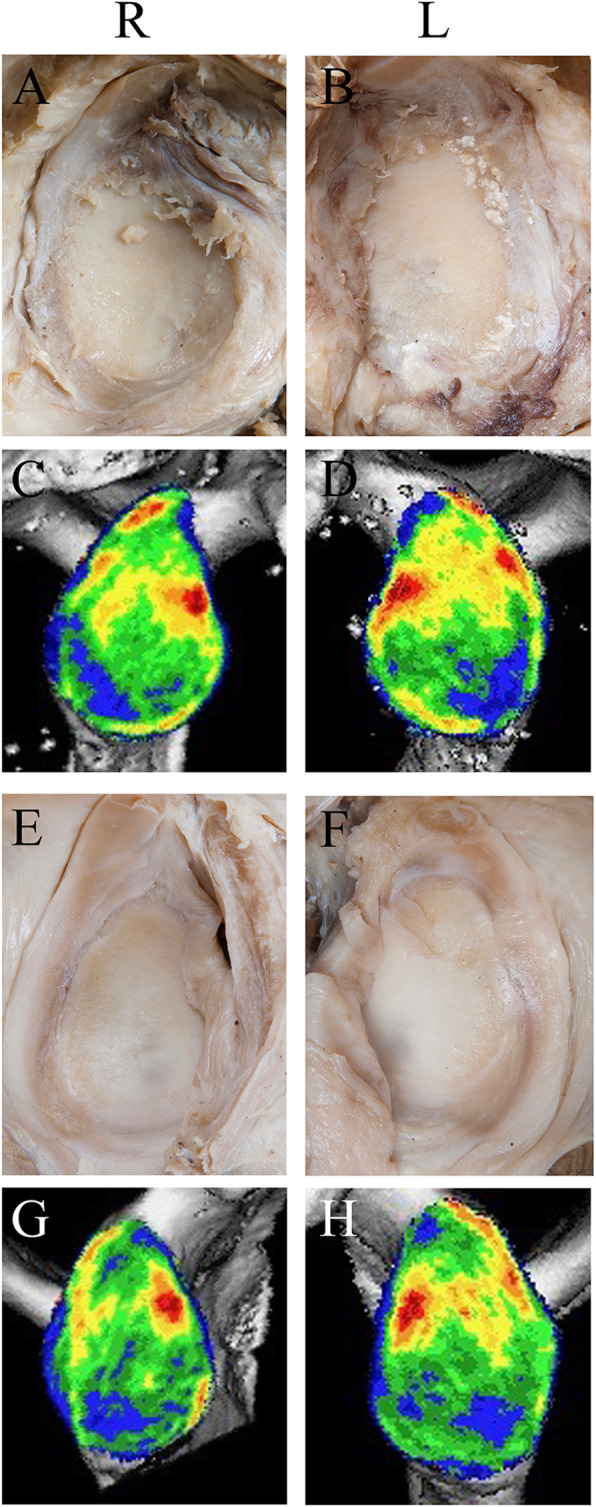
Mineralisation distribution in glenoids with inclination angles < 10°. **A**-**D** 73 years old male (inclination: R 7.8°, L 3.3°; retroversion: R -6.9°, L -6.7°). **C**,**D** On both sides, in addition to anterior mineralisation, density maxima in cranial and dorsal quadrants corresponding to cartilage defects (**A**,**B**). Cartilage thinning in the anterior, more caudal quadrants (**A**,**B**) mirrored by CT-OAM (**C**, **D**). Some spots along the glenoid rim, probably osteophytes. **E**-**H** 72 years old male (inclination: R 7.6°, L 2.9°; retroversion R -1.7°, L − 0.4°). In the left shoulder, note higher mineralisation in cranial quadrants (**H**) and cartilage lesion (**F**). Some spots along the glenoid rim probably osteophytes

In 15/19 left and right glenoids (79*%*), inclination angles within or outside our defined ranges (compare Table 2) were measured in agreement with correspondingly defined mineralisation patterns, i.e., physiological or different. These agreements were bilateral in 13 individuals, and when expanded to 10.7° in a physiological morphology (Fig. 5A,C), raised unilateral to 16/19 right glenoids (84.2%). In the remaining three cases of differences, inclination angles within our physiological range were combined with cranially enlarged mineralisation areas (Fig. 7C,G) whereby morphological alterations were detected in both, cranial (Fig. 7A) and caudal (Fig. 7A,E) quadrants.

On the left, an inclination of 15.9° was detected in a glenoid with physiological mineralisation distribution and morphology (Fig. 5B,D), and bilateral radiological osteoarthritis grade 0 (data not shown). Three glenoids with inclinations angles within our range defined as physiological, showed mineralisation distributions outside the physiological pattern, one caudal (data not shown) and two cranial (Fig. 7D). Enlarged mineralisation areas were located to the neighbourhood of damaged cartilage, particular on the glenoid rim (Fig. 7D,H), while areas of comparably reduced mineralisation (Fig. 7D,H) were co-localised with destroyed areas (Fig. 7B,F). Some spots were completely congruent between morphology and CT-OAM.

### Comparison of ante−/retroversion angles and CT-OAM

To a lesser extent, positive results (compare Table 2) were found for ante−/retroversion angles in 13/19 (69%) right glenoids, with expanded margin in 15/19 (79%). The four cases of disagreement were either due to retroversion (− 2.1°) in a physiological mineralisation distribution, or to retroversion within the physiological range, but mineralisation different from our physiological pattern, in one case with even distribution, but some spots on the rim and mild signs of osteoarthritis (Fig. 5A, C), and in two cases osteoarthritis grade III (data not shown).

In 11/19 left shoulders (57.9%), positive results (compare Table 2) were found, when expanding the range (− 9°), in 13/19 (68.4%). The six cases of disagreement were due to one retroversion angle within our defined range in a glenoid with dorsally enhanced mineralisation and grade II osteoarthritis (data not shown). Angles outside the physiological range were measured in five glenoids with physiologic mineralisation distribution with corresponding morphology and predominantly low radiological osteoarthritis grades, respectively.

### Determination of optimal ranges for inclination and ante−/retroversion angles

**Fig. 8:**
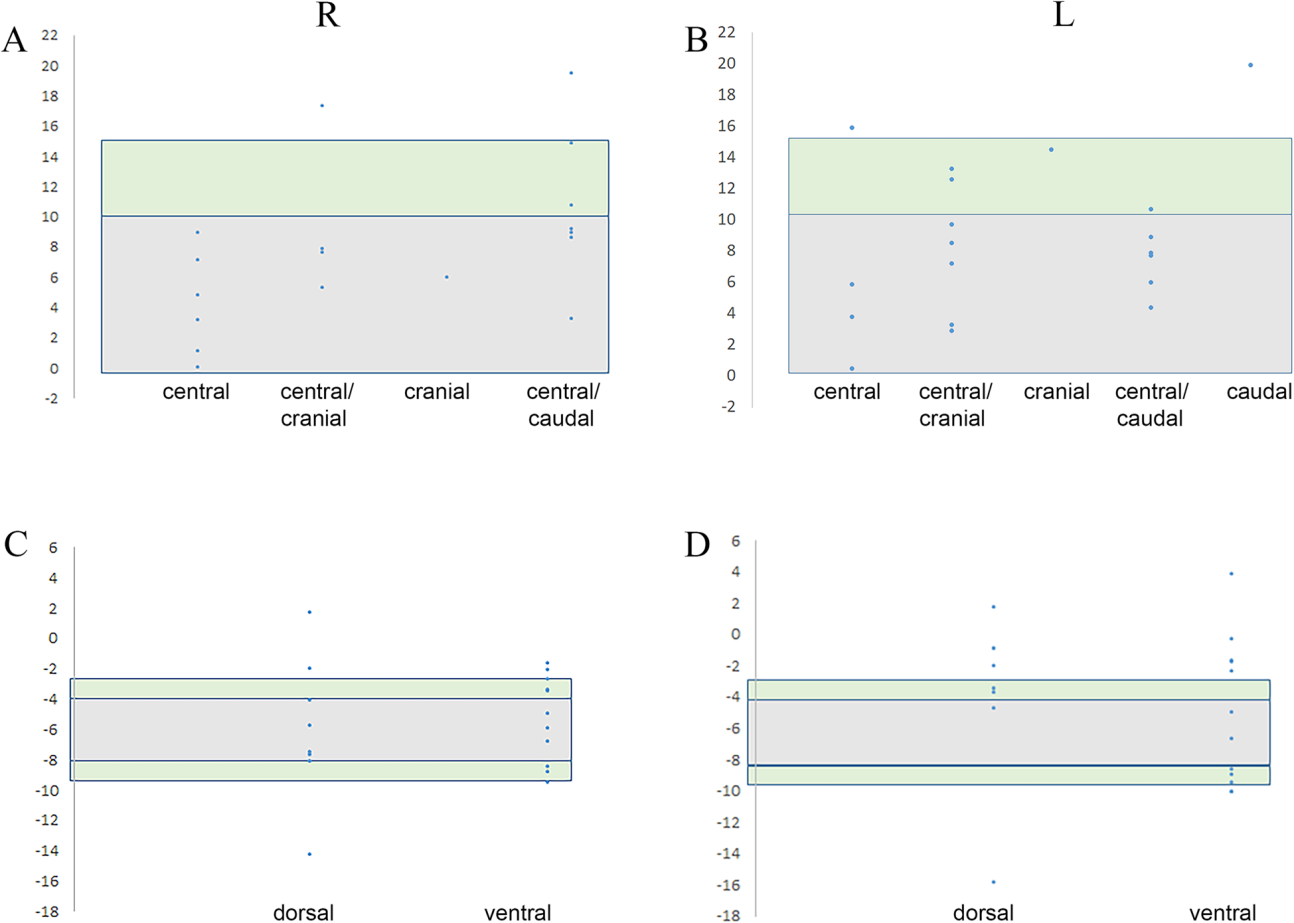
Search for optimal ranges of glenoid angles. Inclination (**A**, **B**) and ante−/retroversion (**C**, **D**) angles (°, Y-axis), covering (grey area) a maximum of physiological mineralisation distribution patterns (x-axis), i.e. predominantly central (**A**,**B**), ventral > dorsal (**C**,**D**), and a minimum of different patterns. **A**,**B** Grey area covers superior inclination ≤10°, green areas expanded margins ≤15° in selected cases. **C**,**D** Grey area covers ante−/retroversion angles from − 8° to − 4°, green areas expanded margins ±1° in selected cases. R right, L left shoulders

Based on the above results, a range of angles was searched covering on the one hand, a maximum of physiological mineralisation patterns (see Figs. 4A, 5D) as published [28, 45], and on the other hand, a minimum of different patterns (see Table 2). In cases of morphological osteoarthritic appearance (Fig. 6B,D), the original range for inclination ≤10° was applied. By iterative recalculations of test ranges, a superior inclination of ≤10° was defined as optimal (Fig. 8A,B), which is within the order of magnitude of current literature, e.g., ([12, 15, 24, 35, 53], with further references). Selected cases at the margins of this range were individually inspected for healthy appearance (e.g., Fig. 5), and the proposed range expanded to ≤15° for recalculation. For ante−/retroversion angles, the test range was defined between − 8° to − 4° (Fig. 8C,D) in the order of magnitude of current literature [15, 19, 24, 27, 35, 48]. Selected cases at the margins of this range were inspected, and if suitable, the proposed range was expanded by ±1°.

## Discussion

In this interdisciplinary study, we examined shoulder specimens from ten female and nine male body donors with an average age of 81.5 years, which is representative for the elder population like our previous study [35]. Also the larger glenoids in males than females were comparable to preceding studies in Middle Europe and America including our own [35] whereby in the present study, glenoid sizes were moderately larger. Also humeral head size was similar to the normal cohort in a previous study [26], which attributed their higher values achieved to improved accuracy of 3D-CT-reconstruction. Thus, our study cohort is comparable to recent studies using 3D-CT, however, data of 19 donors are a limitation.

The average inclination angle in male and female donors combined was 8.4°, which is within the order of magnitude of current literature, e.g., ([15, 24, 31, 35, 53], with further references). In our previous study on a similar cohort [35], mean inclination of 13° was similar to 12° in predominantly female postoperative patients [16]. Our current findings are similar to osteoarthritic individuals (7.6°) in a study comparing pathologies [8] with higher inclination angles (13.6°) in massive rotator cuff tears. Based on the literature (see above), we found inclination angles ≤10° in glenoids with mineralisation distribution patterns considered as healthy [45], frequently combined with healthy morphology and low 3D-CT osteoarthritis gradings. In agreement, high inclination was recently grouped as ≥10°, low inclination between 0° to 10° and no inclination ≤0° [39].

Simon and co-workers [46] used CT-OAM to analyze glenoid component loosening in total shoulder arthroplasty. In concentric glenoids, mineralisation was homogeneously distributed, with greater mineralisation in the central zone, whilst in the eccentric group, mineralisation distribution was inhomogeneous, predominantly in the posterior followed by the inferior zone. The authors concluded that CT-OAM may be effective to assist in preoperative planning for shoulder arthroplasty. In agreement, in three cases of inclination angles within our range, enlarged areas of mineralisation were found in the neighbourhood of damaged cartilage, particular on the glenoid rim, and diminished mineralisation within destroyed area, but also congruent spots were found. Enlarged areas of mineralisation as a sign of enhanced workload in cranial parts accompanied by morphological destruction in the caudal quadrants may be explained by pain avoidance. More data are required on pathological shoulder specimens, however first indication exists that CT-OAM may be suitable to announce early arthritic processes.

In the present study, we selected retroversion angles within a very narrow range, which may be the reason why they were more rarely found in glenoids with physiological mineralisation patterns. The mean ante−/retroversion angles from male and female donors combined were − 5.2°, which is similar to − 4° in the control group in another study [43] using the Friedman method [13], and to − 3.8° [6], and − 8.5° using 3D-CT-reconstruction [10]. In a study comparing a new 2D method, retroversion angles of − 19° were measured in the control group, whilst measurements according to Friedman revealed retroversion of − 1° [41], similar to our previous study [35]. Thus, current findings point to a retroversion between − 8° to − 4° with a tolerance of ±1° as physiological biomechanical situation, which is similar to previous work [15, 19, 43, 48]. During surgery, 10–15° of retroversion can be corrected using eccentric reaming of the glenoid according to Stephens et al. [48]. Similarly, Mizuno et al. [36] suggested a limit of − 10° as surgical aim for correction using eccentric reaming. More data are required, also whether the range of the present study could be expanded, e.g. until − 10° to − 3°, particularly in females. Data base is very limited, however, in both females with retroversion angles of − 10.1°, mineralisation distribution was physiological and osteoarthritis graded 0 and II, but in glenoids with higher retroversions in another female (− 14.3°, − 15.9°), dorsally enhanced mineralisation and osteoarthritis grades III and IV were found. We hypothesise whether the smaller glenoids in females may predispose to tolerate moderately higher retroversions. Nevertheless, higher retroversion angles predispose for dorsal shoulder subluxation and glenoid loosening [15], whereby Walch and co-workers [50] found retroversion of 24° (versus 17.4°) to predispose for glenoid loss.

The data set has to be looked at with caution due to several limitations. First of all, the study was conducted on 19 body donors only. Further limitations of the study are that morphological evaluations are predominantly subjective and strongly rely on the experience of raters. Inter- and intra-observer variabilities were not measured. Furthermore, our post-mortem evaluation did not include functional changes or clinical reports. Since other omarthritis grading systems either included input from other radiological methods or functional examinations, we introduced our radiological omarthritis grading system as combination and adaptation of the Kellgren and Lawrence Score [25], the Samilson and Prieto classification [44], and the Larsen classification [30] since these are independent of aetiology and potential post-mortem changes. Elsharkawi and co-workers [11] recommended expansion of the commonly used Samilson and Prieto classification [44] especially for clinical and scientific purposes so that one advantage of our grading system is that it expands the primarily used Kellgren and Lawrence system [25] with respect to radiological signs including osteophyte formation and joint narrowing, but also includes inferior humeral head and glenoid exostosis as well as destructive abnormality and signs of erosions and allows for a more precise grading. A limitation of our system, however, is that due to 3D-CT it does not include minor changes in the cartilage, as they are typical for initial stages of osteoarthritis, however this was partially compensated by the morphological investigations of the dissected specimens. In the present study, the critical shoulder angle CSA [4, 37] was outside the original scope of this work. Meanwhile for osteoarthritis, the inclination angle is proposed to be of lesser relevance than CSA [1, 53], whereby among the three components of CSA, glenoid inclination seems to have the largest impact regarding joint stability [5].

## Conclusion

Although the data set is limited, in this elderly sex-balanced study cohort, the superior inclination angle between 0° to 10°-15° and retroversion between − 9° to − 3° was associated with physiological mineralisation distribution of the glenoid. Enlarged areas of mineralisation appeared more frequently combined with discrete changes of the cartilage, but pronounced cartilage defects with decreased mineralisation and generally elevated mineralisation in the surrounding. More experience is required to expand the data set to other pathological shoulder specimens, however first indication exists that CT-OAM may be suitable to announce early osteoarthritic processes.

## Supplementary Information

**Additional file 1: Supplement 1** Sizes of glenoid and humeral head (millimetres, mm) measured by 3D-CT using landmarks depicted in Fig. 3. SD: Standard deviation.

## Data Availability

The datasets analyzed during the current study are available from the corresponding author on reasonable request.
