# Supplementary material for: CT-based and morphological comparison of glenoid inclination and version angles and mineralisation distribution in human body donors
Source: BMC Musculoskelet Disord. 2021 Oct 5;22:849. doi: 10.1186/s12891-021-04660-4 (PMC8493698; doi:10.1186/s12891-021-04660-4)
Supplement: Supplementary file 1 — Additional file 1: Supplement 1 Sizes of glenoid and humeral head (millimetres, mm) measured by 3D-CT using landmarks depicted in Fig. 3. SD: Standard deviation. [file 12891_2021_4660_MOESM1_ESM.docx]

**Supplement 1** Sizes of glenoid and humeral head (millimetres, mm) measured by 3D-CT using landmarks depicted in Fig. 3. SD: Standard deviation.

| **Measurement** | **Landmarks** | **Average  (mm)** | **SD** | **Range (mm)** |
| --- | --- | --- | --- | --- |
| Glenoid height | A-B | 38.4 | 3.2 | 31.6-43.3 |
| Glenoid width | C-D | 29.5 | 3.0 | 24.1-34.5 |
| Humeral head height | A-B | 48.8 | 4.3 | 41.2-57.2 |
| Humeral head width | C-D | 45.1 | 3.8 | 38.8-50.5 |
| Humeral head depth | E-F | 21.2 | 2.7 | 16.5-26.1 |

Sizes of glenoid and humeral head presented from male and female body donors combined. Landmarks depicted as reference points in Fig. 3A-C.

|  | **Sex** | **N** | **Mean (mm)** | **SD (mm)** | **Range (mm)** |
| --- | --- | --- | --- | --- | --- |
| Glenoid height | m | 18 | 41.3 | 0.9 | 40.0-43.3 |
|  | f | 20 | 35.7 | 1.7 | 31.6-38.2 |
| Glenoid width | m | 18 | 32.0 | 2.0 | 28.0-34.5 |
|  | f | 20 | 27.3 | 1.6 | 24.1-30.3 |
| Humeral head height | m | 18 | 52.5 | 2.5 | 47.6-57.2 |
|  | f | 19 | 45.3 | 2.3 | 41.2-50.0 |
| Humeral head width | m | 17 | 48.0 | 1.5 | 44.4-50.5 |
|  | f | 15 | 41.8 | 2.6 | 38.8-48.2 |
| Humeral head depth | m | 18 | 22.7 | 1.9 | 19.0-25.6 |
|  | f | 20 | 19.8 | 2.7 | 16.5-26.1 |

Sizes of glenoid and humeral head sorted by sex. N=number of specimens measured.
